# Supplementary material for: Lumican Inhibits In Vivo Melanoma Metastasis by Altering Matrix-Effectors and Invadopodia Markers
Source: Cells. 2021 Apr 8;10(4):841. doi: 10.3390/cells10040841 (PMC8068222; doi:10.3390/cells10040841)
Supplement: Supplementary file 1 [file cells-10-00841-s001.pdf]

B16F1 snail

total FAK

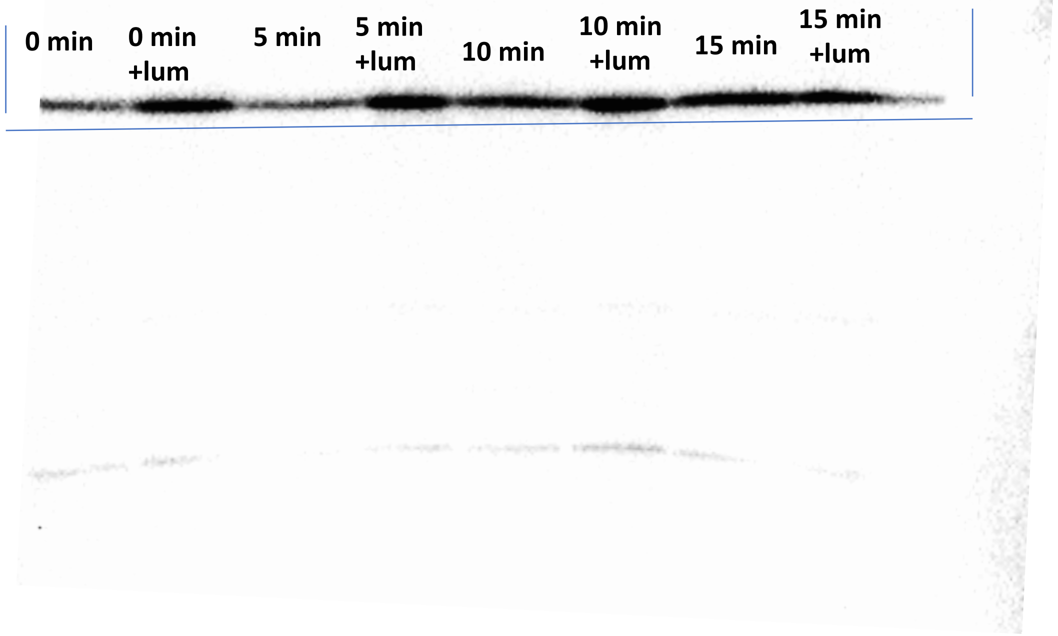

pFAK

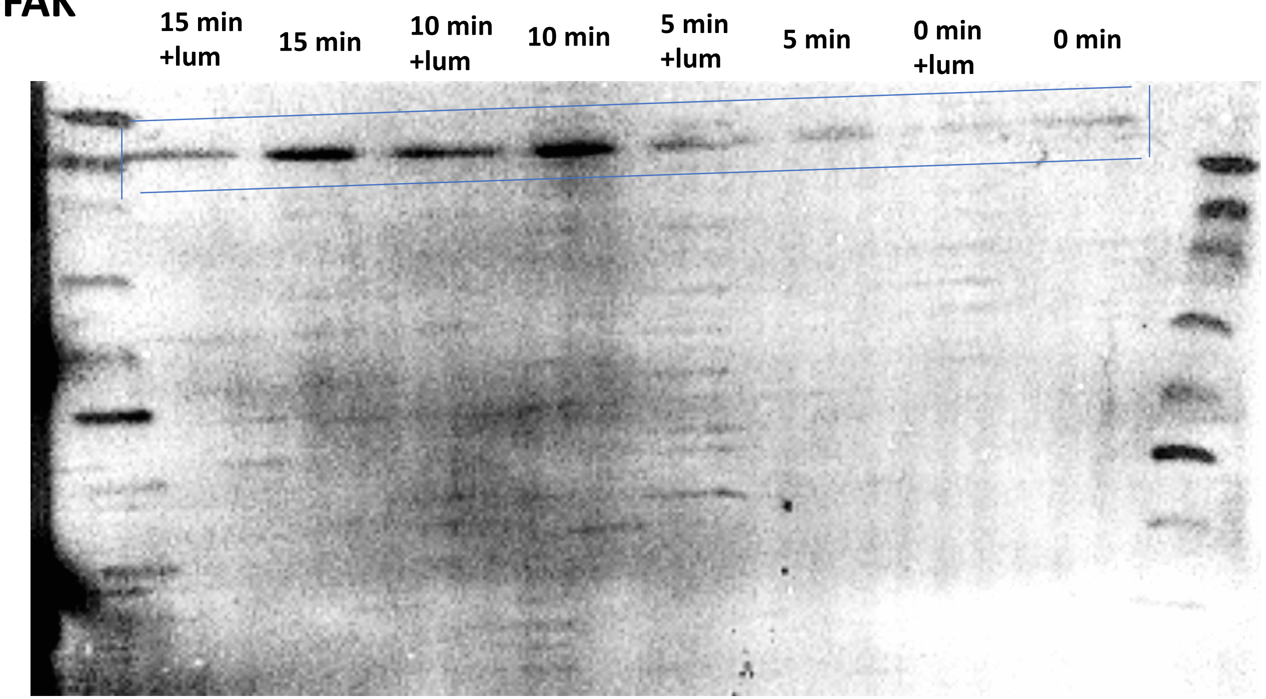

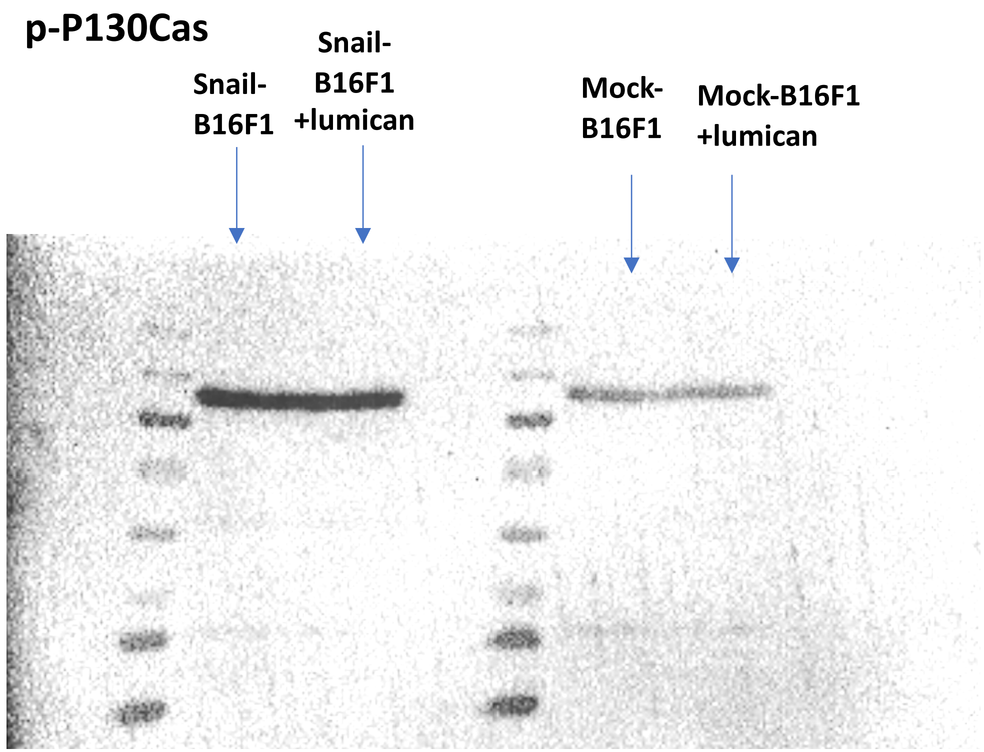
p-P130Cas
Snail-
B16F1
Snail-
B16F1
+lumican
Mock-
B16F1
Mock-B16F1
+lumican

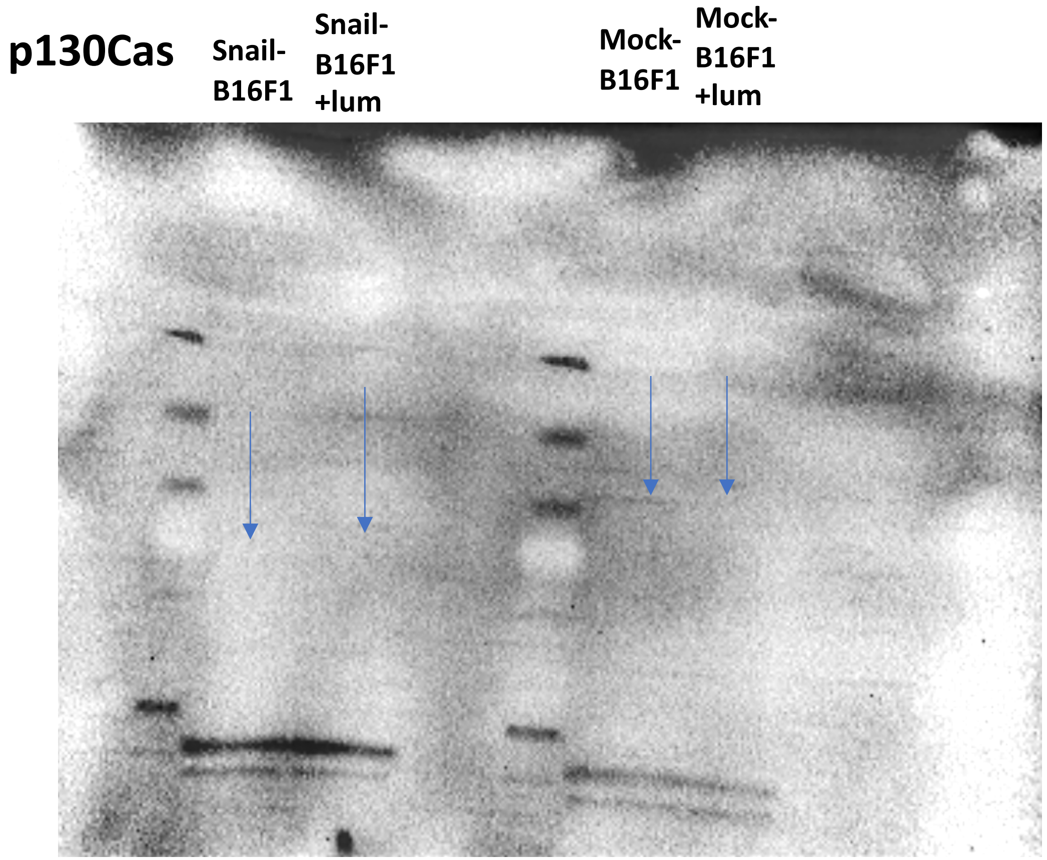
p130Cas
Snail-
B16F1
Snail-
B16F1
+lum
Mock-
B16F1
Mock-
B16F1
+lum

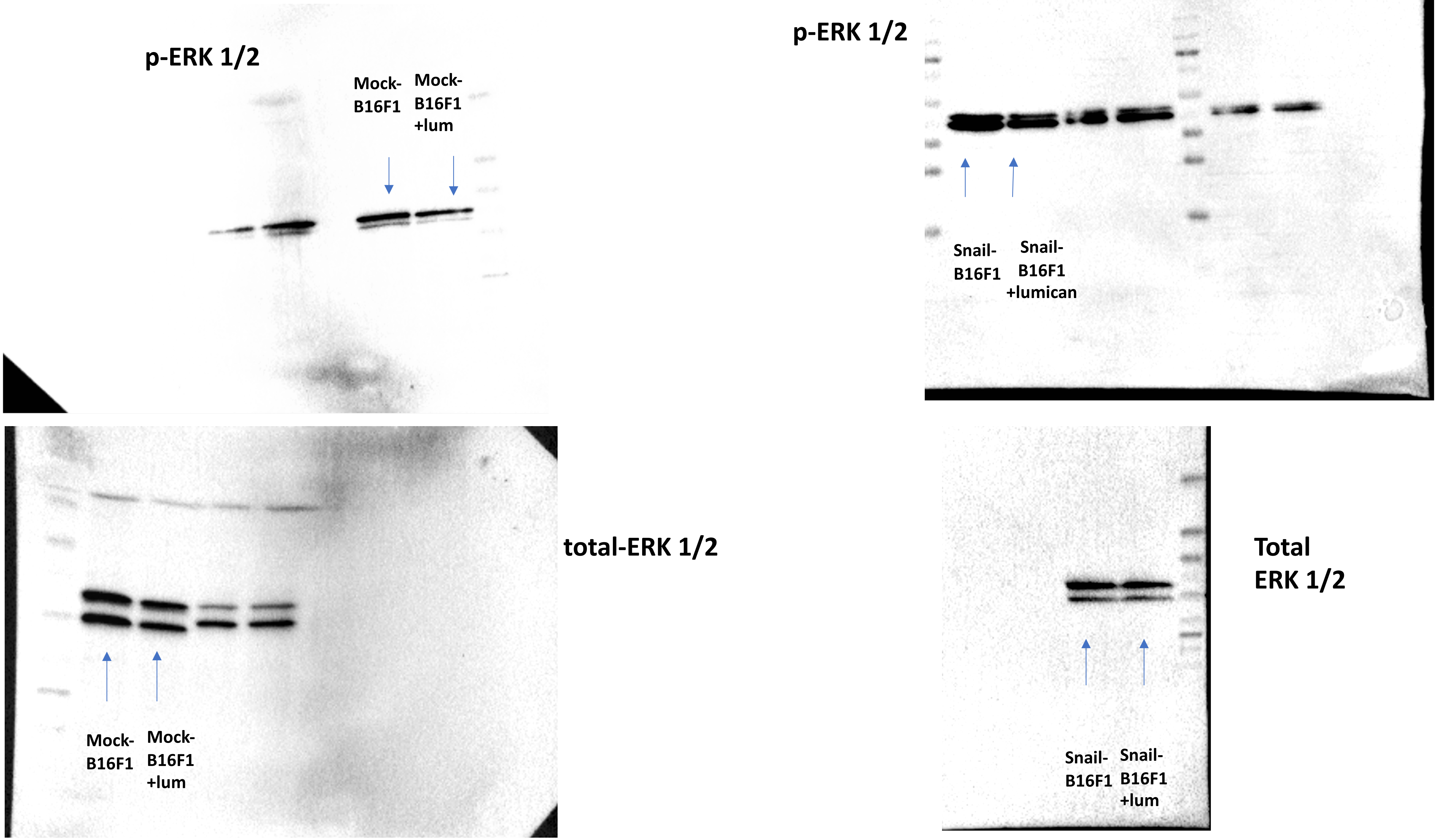
p-ERK 1/2
Mock-B16F1
Mock-B16F1 +lum
p-ERK 1/2
Snail-B16F1
Snail-B16F1 +lumican
total-ERK 1/2
Mock-B16F1
Mock-B16F1 +lum
Total ERK 1/2
Snail-B16F1
Snail-B16F1 +lum

pAKT

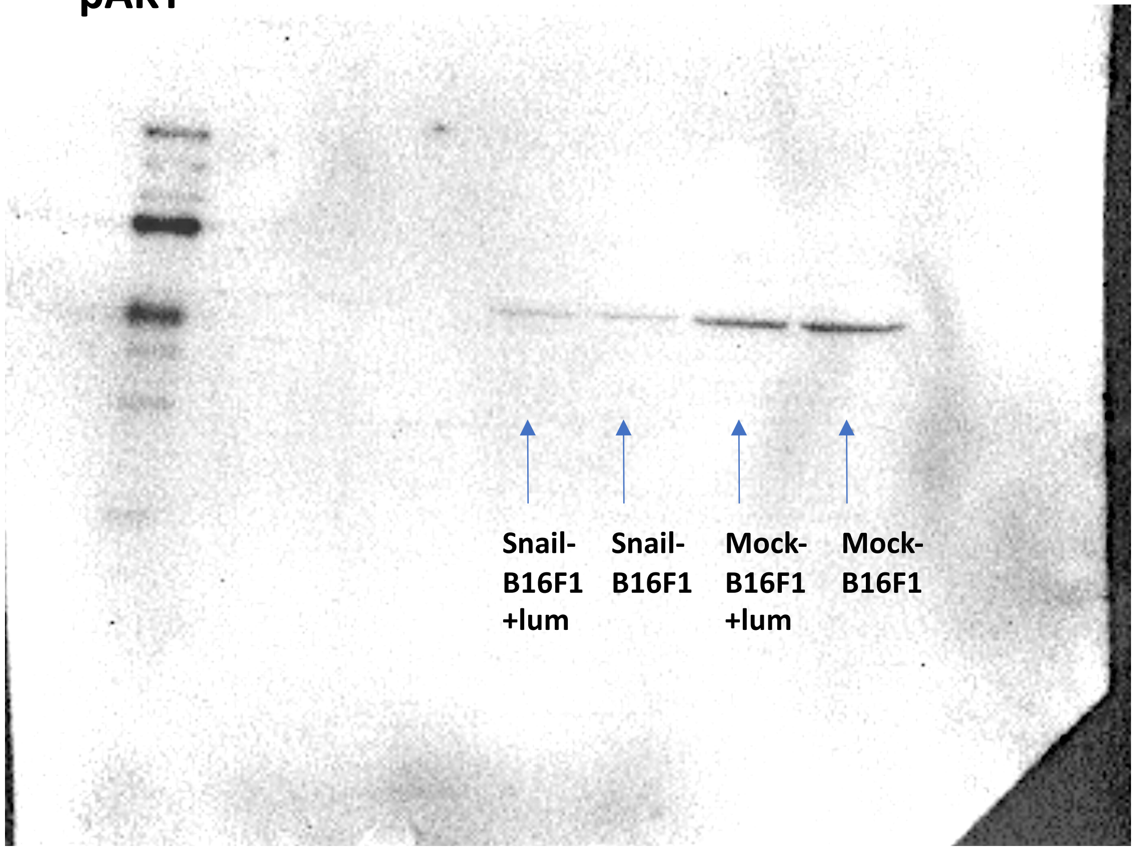

Total AKT

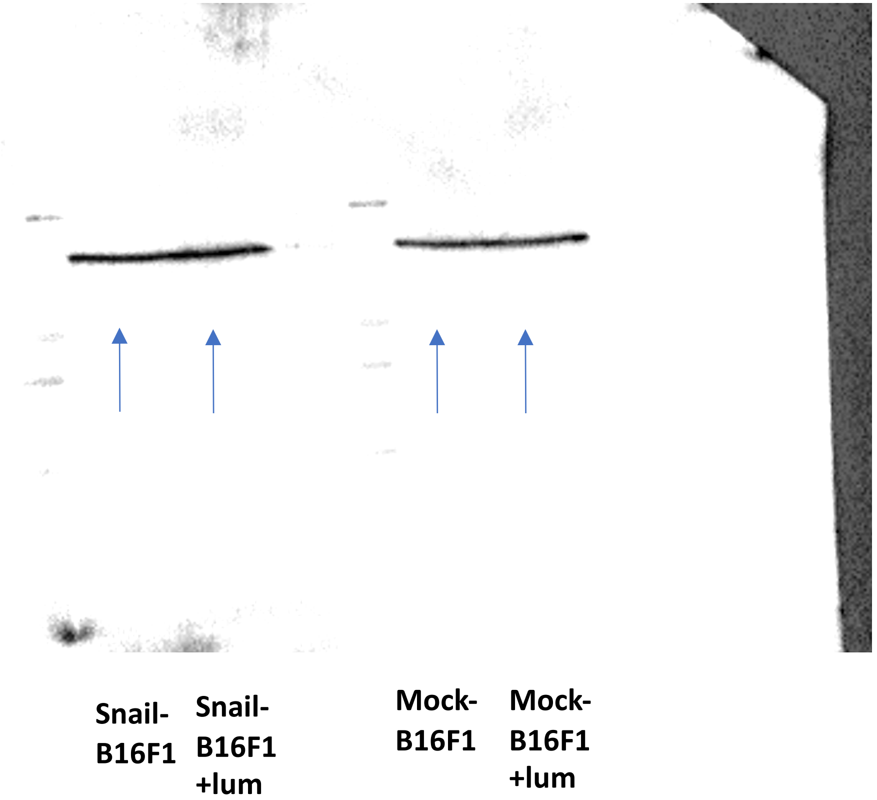

Total GSK3 a/b

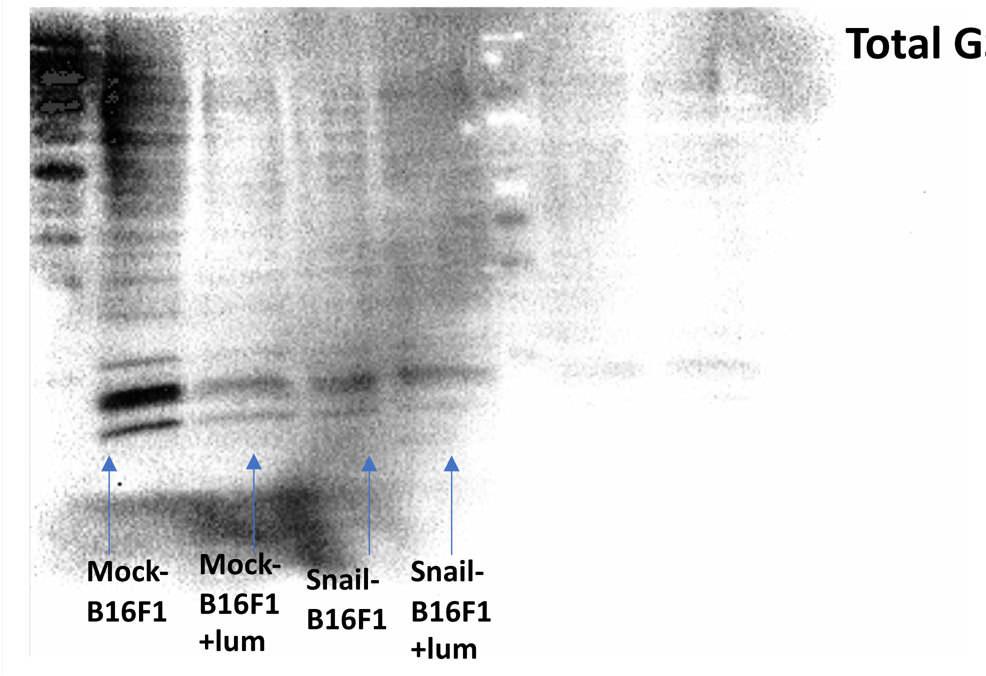

p-GSK3 a/b

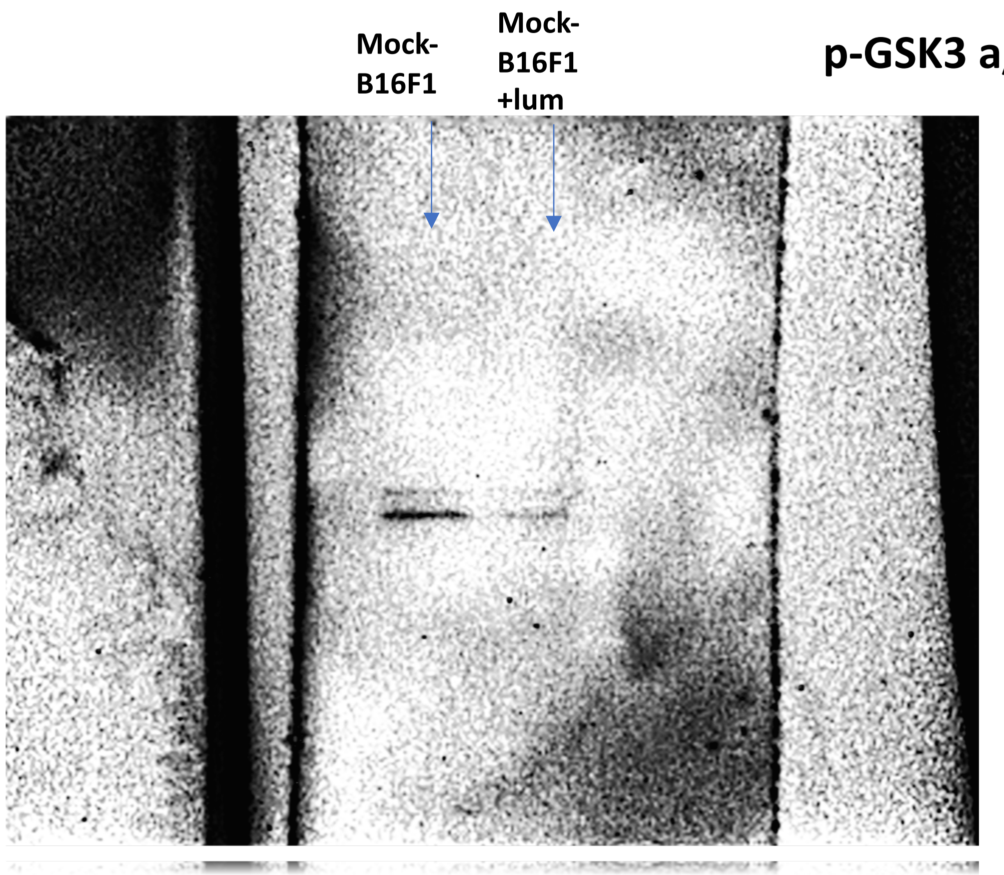

p-GSK3 a/b

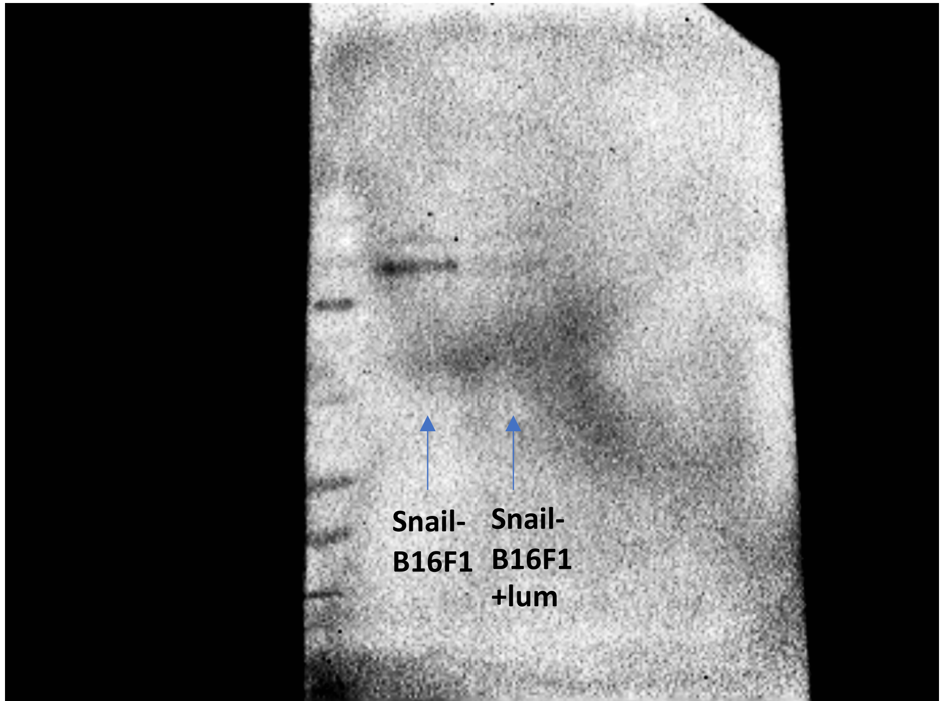

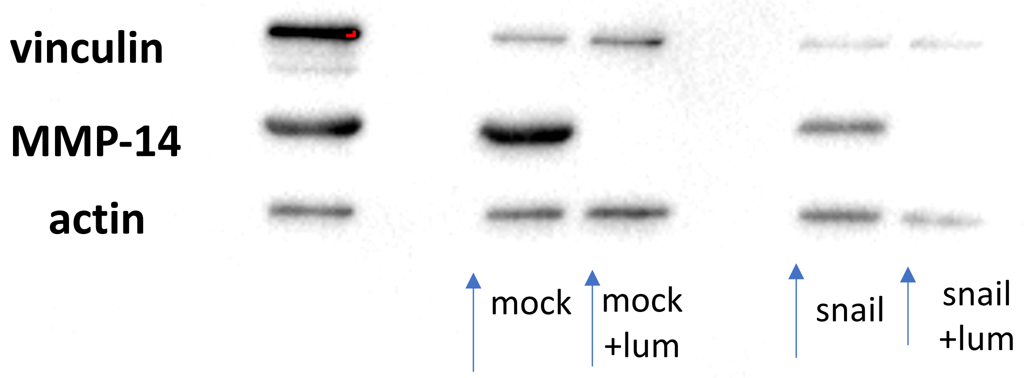
vinculin
MMP-14
actin
mock
mock
+lum
snail
snail
+lum

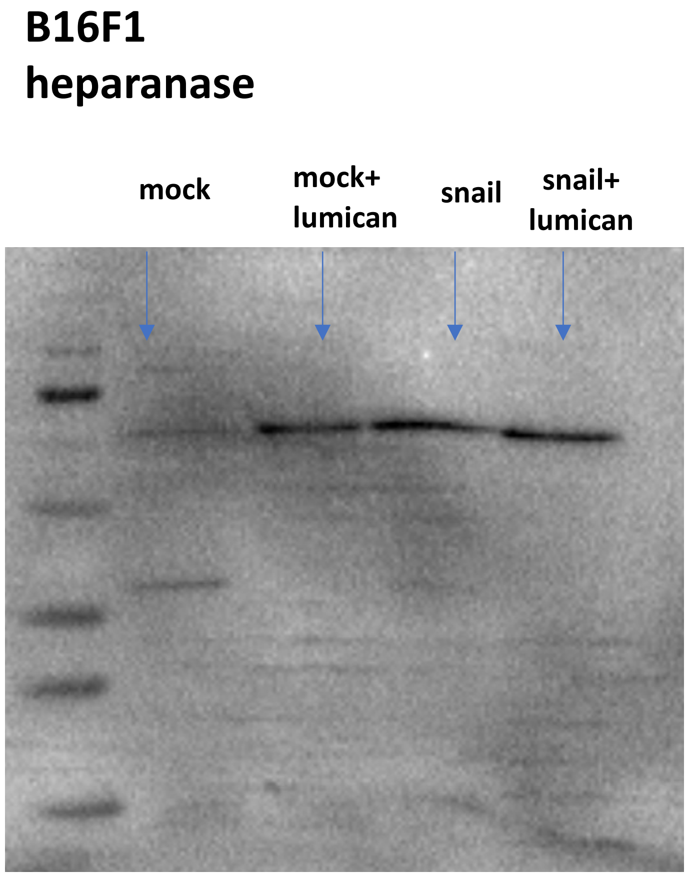
B16F1
heparanase
mock
mock+
lumican
snail
snail+
lumican

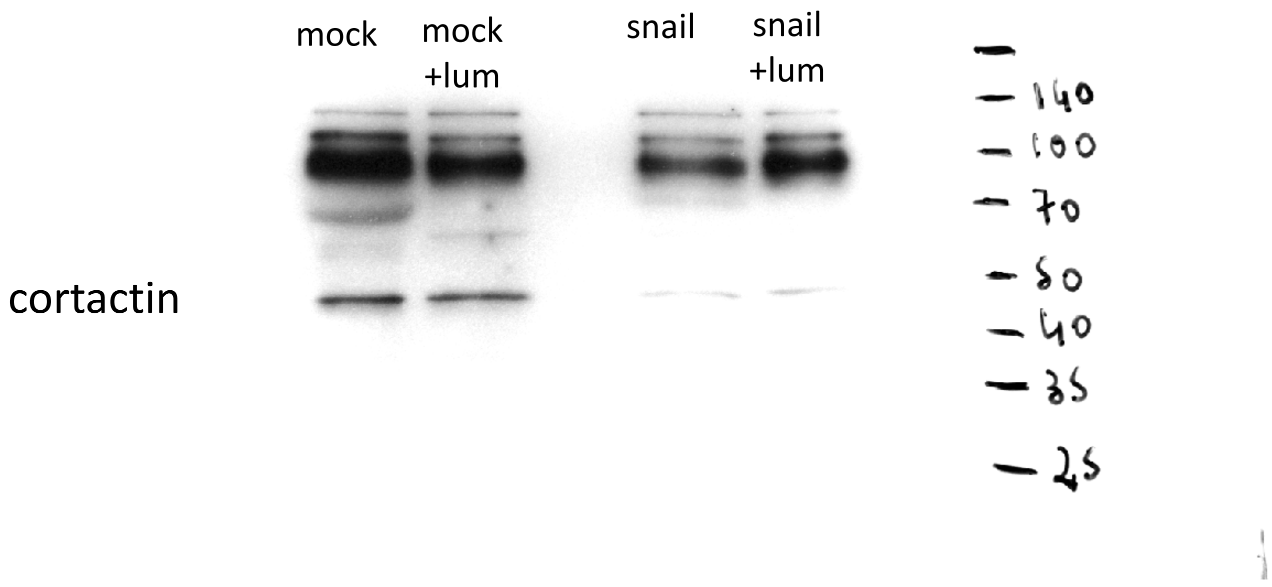
mock
mock +lum
snail
snail +lum
cortactin
140
100
70
50
40
35
25
